# Salvianolic Acid B Inhibits Activation of Human Primary Hepatic Stellate Cells Through Downregulation of the Myocyte Enhancer Factor 2 Signaling Pathway

**DOI:** 10.3389/fphar.2019.00322

**Published:** 2019-04-11

**Authors:** Wenwei Zhang, Jian Ping, Yang Zhou, Gaofeng Chen, Lieming Xu

**Affiliations:** ^1^ Shuguang Hospital Affiliated to Shanghai University of Traditional Chinese Medicine, Shanghai, China; ^2^ Institute of Liver Diseases, Shanghai University of Traditional Chinese Medicine, Shanghai, China; ^3^ Shanghai Key Laboratory of Traditional Chinese Medicine, Shanghai, China; ^4^ Key Laboratory of Liver and Kidney Diseases, Ministry of Education, Shanghai, China; ^5^ Yueyang Hospital Affiliated to Shanghai University of Traditional Chinese Medicine, Shanghai, China

**Keywords:** myocyte enhancer factor 2, hepatic stellate cell, TGFβ1, salvianolic acid B, liver fibrosis

## Abstract

Various isoforms of myocyte enhancer factor 2 (MEF2) have been shown to play a role in the activation of rat hepatic stellate cells (HSCs) in culture. The signals that regulate MEF2 in HSCs are unknown. In addition, whether MEF2s regulate the activation of human HSCs (H-HSCs) is unclear. Here, we studied the expression and function of MEF2s in H-HSCs. Our data showed that the levels of MEF2A, C, and D proteins were high in liver tissues from patients with cirrhosis and increased during culture-induced activation of primary H-HSCs. Exposure of H-HSCs to transforming growth factor beta 1 (TGF-β1) led to a significant increase in MEF2A and C protein levels and enhanced MEF2 activity. Interestingly, TGF-β1 did not further enhance MEF2D levels. Furthermore, TGF-β1 activated p38 mitogen-activated protein kinase (MAPK) and led to increased phosphorylation of MEF2C at its p38 recognition site. Inhibition of p38 MAPK inhibited both TGF-β1- and culture-induced activation of MEF2. The activity of collagen I reporter in H-HSCs was significantly reduced when MEF2A and MEF2C were blocked with overexpression of dominant negative MEF2 mutants. Salvianolic-acid B (SA-B), a water-soluble element of *Salvia miltiorrhiza* known to have anti-fibrosis effects, attenuated both basal and TGF-β1-induced increased levels of MEF2A and C mRNA and protein. In addition, SA-B inhibited MEF2 activity, which correlated with reduced expression of the HSC activation markers, α-smooth muscle actin (α-SMA), and collagen I. Administration of SA-B reduced MEF2A *in vivo*, which was accompanied by reduced levels of α-SMA in a model of dimethylnitrosamine-induced rat liver fibrosis. We concluded that the MEF2 transcription factor was stimulated by TGF-β1 in H-HSCs. Antagonizing TGF-β1-induced activation of the MEF2 signaling pathway may account in part for the anti-fibrosis effects of SA-B.

## Introduction

Hepatic stellate cells (HSCs) are the main cells responsible for liver fibrosis. Activation of HSCs, which involves the transition of quiescent cells into highly proliferative, fibrogenic, and contractile myofibroblasts, represents the final common pathway of the hepatic response to liver injury induced by various factors. Transforming growth factor beta1 (TGF-β1) activates HSCs and is considered the most potent cytokine to perpetuate the fibrogenic response in the liver (Friedman, 2008; Tsuchida and Friedman, 2017). The pro-fibrogenic effects of TGF-β1 are mediated through several key downstream effectors. These primarily include p38 mitogen-activated protein kinase (MAPK), extracellular signal-regulated kinase 1 and 2 (ERK1, 2), and Smad signaling pathways (Zhang, 2018).

Interestingly, many studies suggest that HSCs isolated from rats and humans, and myofibroblasts, their activated counterparts, in particular, express a number of neuronal and muscle cell markers, such as neurotrophins, neurotrophin receptors, brain-derived nerve growth factor, MyoD, and α-smooth muscle actin (α-SMA) (Trim et al., 2000; Geerts, 2001; Vincent et al., 2001; Cassiman et al., 2002). This suggests that factors involved in the regulation of neuronal and muscle cells may also play a role in HSCs. Indeed, our previous studies have shown that the transcription factor, myocyte enhancer factor 2 (MEF2), represents a class of proteins known to play key roles in neurons and muscle cells and participates in the activation of rat HSCs in culture (Wang et al., 2004). Four mammalian isoforms of MEF2, A–D, have been identified. The MADS and MEF2 domains present at the N terminus are highly homologous among MEF2s and are responsible for mediating dimerization among them and DNA binding. The C-terminus regions of MEF2 proteins are required for transcriptional activation (Black and Olson, 1998). Many important signaling pathways converge on MEF2. These include p38 MAPK, Smads, and calcium/calmodulin/calcineurin pathways (Mao and Wiedmann, 1999; Quinn et al., 2001; Olson, 2004; Wales et al., 2014). The p38 MAPK signaling pathway has been shown to regulate MEF2 transcriptional activity in various cell types and to promote neuronal survival and differentiation (Phiel et al., 2001; Illi et al., 2005; Ju et al., 2005; Ramachandran et al., 2008). However, whether and how MEF2 is regulated by TGF-β1 during liver fibrosis is not entirely clear.

Great efforts have been made to understand the molecular mechanisms that underlie HSC activation during liver fibrosis. Currently, much of our knowledge of how stellate cells behave during liver injury has been gained through studies using animal model systems (Tsuchida and Friedman, 2017). However, whether and to what degree the mechanisms identified in animal systems operate in (H-HSCs) is not entirely clear and requires validation in order to establish their relevance to human disease. Therefore, we studied the regulation of MEF2 in H-HSCs. Our data revealed high levels of MEF2 expression in human cirrhotic liver tissues and freshly isolated H-HSCs. Expression of MEF2 was activated by TGF-β1 *via* p38 MAPK.

Salvianolic acid B (SA-B) is an effective water-soluble component of *Salvia miltiorrhiza*. Previous research indicates that SA-B inhibits the synthesis of collagen I (Col I) in LX-2 cells independently of TGF-β1 stimulation, and the anti-fibrotic effect of SA-B is due to direct inhibition of p38 signaling and inhibition of the cross-talk between Smad and ERK signaling (Lv and Xu, 2012). In another study, we found that SA-B lowers portal pressure in rats with dimethylnitrosamine (DMN)-induced cirrhosis *in vivo*. In addition, SA-B attenuates endothelin-1 (ET-1)-induced HSC contraction by inhibiting the activation of RhoA and Rho-associated coiled coil-forming protein kinase (ROCK) II and downstream MYPT1 phosphorylation at Thr^696^ *in vitro* (Xu et al., 2012). The present study indicates that SA-B inhibits MEF2 activity in H-HSCs and attenuates its levels in a liver fibrosis model in the rat.

## Materials and Methods

### Materials

Human liver specimens were obtained from patients who had undergone liver resection or transplantation for liver carcinoma or end-stage chronic liver diseases, following the guidelines and with the approval of the institution. The study was approved by the Ethics Committee of Shuguang Hospital. All subjects gave written informed consent in accordance with the Declaration of Helsinki. Salvianolic acid B (SA-B), an effective water-soluble element of Radix *Salvia miltiorrhiza*, was purified and provided by Shanghai Institute of Materia Medica, Chinese Academy of Sciences. Polyclonal antibodies for MEF2A and monoclonal antibodies for p-38, p-ERK1/2, and ERK1 were purchased from Santa Cruz Biotechnology (TX, United States); polyclonal antibodies for MEF2C and p-p38, from Cell Signaling; polyclonal antibodies for collagen I, from Calbiochem (CA, United States); monoclonal antibody for MEF2D, from BD Biosciences (MD, United States); and monoclonal antibodies for β-actin and α-SMA from Sigma-Aldrich (St Louis, MO, United States). Inhibitors of ERK (PD98059) and p38 MAPK (SB203580) were purchased from Calbiochem (CA, United States).

### Isolation and Culture of HSCs

The HSCs were isolated as described previously (Zhang and Xu, 2007). Briefly, H-HSCs from ~50 g of liver tissue and HSCs from adult male Sprague–Dawley rats (~400 g) were isolated with pronase (Roche, Germany)/collagenase (Serva, Germany) treatment, followed by density gradient centrifugation using Nycodenz (Axis-shield, Norway) solution to isolate the buoyant and lipid rich stellate cells. Isolated HSCs were cultured in medium 199 (Gibco) containing 10% fetal calf serum. When the cultures reached confluence, they were trypsinized and passaged at a ratio of 1:3.

### Immunofluorescent Staining

The H-HSCs were plated on glass coverslips (Fisher Scientific) in 12-well plates and cultured with 10% fetal calf serum in medium 199. After fixation in methanol/acetone (1:1), cells were washed twice with phosphate-buffered saline and incubated with blocking solution for 1 h. Primary antibodies (Neo Markers) and fluorescence labeled anti-rabbit IgG or anti-mouse IgG were incubated with the cells for 1 h respectively. Cells were mounted on glass slides using the ProLong Antifade kit (p-7481, Molecular Probes, Eugene, OR, USA), viewed, and photographed with a Leica TCS-Sp2 (UV) confocal laser scanning microscope (Leica, Germany).

### RNA Preparation and Reverse Transcription Quantitative Polymerase Chain Reaction (RT-qPCR)

Total RNA was prepared from H-HSCs using the TRIzol total RNA preparation kit, following the manufacturer’s instructions (Gibco). The mRNA expression was measured using SYBR Green Real-time PCR Master Mix (TOYOBO), and the ViiA 7 Real-Time PCR System (ABI, Carlsbad, CA, United States). The MEF2A primers (sequence 5′ to 3′) were as follows, forward: TGC GAC AGC CCA GAC CCT G; reverse: GAG GTG GCA GAC CAG GTG CG; and MEF2C primers (sequence 5′ to 3′), forward: CCA GTA TGC CAG CAC CGA C; reverse: CGT CTC CAC GAT GTC TGA G. The housekeeping gene β-actin was used as a reference gene for normalization.

### Cellular Protein Extracts and Western Blot

Cytoplasmic and nuclear extracts were prepared from H-HSCs (NUC-101, Sigma) and protein concentrations of the supernatant were measured using a protein concentration kit (DC Protein Assay; Bio-Rad) according to the manufacturer’s instructions. All western blot analyses were repeated at least three times. In general, data from a representative experiment are shown.

### Luciferase Reporter Gene Assay

The MEF2 and collagen α1 (I) luciferase reporter assays were performed as previously described (Gong et al., 2003). The H-HSCs were transfected with either an MEF2 luciferase reporter gene (MEF2 binding site underlined, wild-type: TCGACGGGCTATTTTTAGGGCC; mt: 5-TC-GACGGGC*G*ATTTTT*C*GGGCCG-3 (Shin et al., 1999), the italics indicate mutated sites) or a collagen I (Rippe et al., 1997) promoter construct, along with a plasmid encoding β-galactosidase (β-Gal) (CMV-β-Gal) by the Lipofectamine 2000 transfection method (Invitrogen) unless stated otherwise. Cellular extracts were assayed for both luciferase and β-Gal activities. Relative fold change in luciferase activity was calculated based on the efficiency of transfection (β-Gal value).

### Induction of Liver Fibrosis in Rats

Male Sprague-Dawley rats (150–160 g) were injected with DMN (10 mg/kg body weight) intraperitoneally for three consecutive days each week. A peritoneal injection of normal saline (N.S). was administered to the normal control group. The SA-B treated group was treated with SA-B (12.5 mg/kg) intragastrically for 3 weeks (Xu et al., 2012). The animal experiments were approved by the Committee on the Care and Use of Live Animals for Teaching and Research of the Shanghai University of Traditional Chinese Medicine.

### Histology and Immunohistochemistry

Portions of liver tissue (approximately 1 × 0.8 × 0.3 cm^3^) were fixed by immersion in 8% buffered methanol and then processed and paraffin-embedded. After deparaffinization, sections of 4-μm thickness were stained with hematoxylin-eosin (H&E) and sirius red. In preparation for immunostaining, paraffin sections of the liver samples were rehydrated and washed for 5 min, three times in Tris-buffered saline. The tissue sections were blocked for 60 min at 37°C. The specimens were then incubated with the primary antibody overnight. The sections were washed in phosphate-buffered saline three times for 10 min and incubated with the secondary antibody for 45 min. The slides were mounted with glycerol.

### Statistical Analysis

Comparisons between groups were performed using the Student’s *t*-test or one-way analysis of variance (ANOVA). *p* < 0.05 was considered statistically significant.

## Results

### Isolation and Phenotypic Characterization of H-HSCs

**Figure 1:**
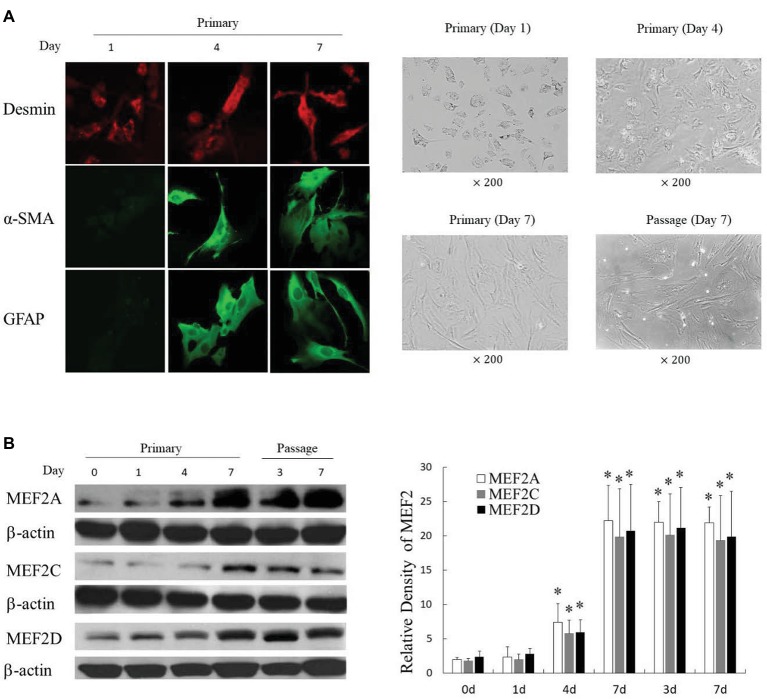
Induction of MEF2 expression during culture-induced activation of primary H-HSCs. **(A)** Expression of activation markers in cultured H-HSCs. The left panel was determined by immunofluorescence. The right panel shows the morphology of H-HSCs under a phase contrast microscope. ‘Day’ indicates the number of days in culture. **(B)** Expression of the MEF2 protein in cultured H-HSCs. The levels of MEF2A, MEF2C, and MEF2D proteins were determined by western blot using isoform-specific antibodies. The right panel shows densitometry values of MEF2 proteins relative to β-actin. Each value represents the mean ± SEM (*n* = 3, *p < 0.05 vs. controls).

We successfully isolated primary H-HSCs. The isolated primary H-HSCs were plated onto uncoated plastic dishes, trypsinized after 7 days, and sub-cultured. Phenotypically, the freshly isolated H-HSCs were round and lipid rich, smaller than hepatocytes and bigger than Kupffer cells. Primary H-HSCs on day 4 in culture had extended pseudopods and assumed a stellar shape. By day 7 in primary culture, the lipid storage in cells was significantly decreased. Passage 1 H-HSCs was proliferated at a faster rate, and these cells developed a fibroblastic morphology and prominent contractile filaments, similar to those observed in rat HSCs (Figure 1A). Immunocytochemical studies have shown that isolated H-HSCs express desmin, a specific marker for HSCs (Wilhelm et al., 2016), throughout the period of culture. In contrast, levels of α-SMA and glial fibrillary acidic protein (GFAP), markers of intermediate filament proteins for activated HSCs (Lim et al., 2008), increased progressively, suggesting that H-HSCs become activated during culture. The high levels of α-SMA and GFAP were maintained following subculture (Figure 1A).

### Expression of MEF2s in Cultured Primary H-HSCs and Freshly Isolated HSCs From Normal and Cirrhotic Human Livers

**Figure 2:**
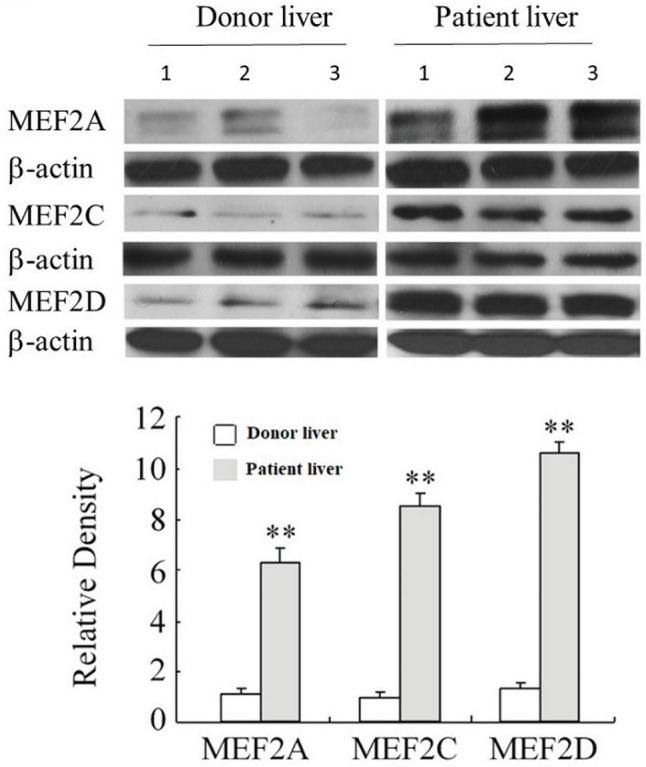
Expression of MEF2 in normal and cirrhotic human livers. The levels of MEF2A, MEF2C, and MEF2D proteins were determined by western blot. Numbers 1 to 3 indicate the samples from individual human. The bottom panel shows densitometry values of MEF2 proteins relative to β-actin. Each value represents the mean ± SEM (*n* = 3, **p < 0.01 vs. controls.).

Examination of MEF2s in cultured primary H-HSCs revealed that levels of MEF2A, C, and D were significantly increased during culture, as H-HSCs became more activated (Figure 1B). The levels of MEF2 proteins were maintained at a high level following subculture. The kinetics of the increase in MEF2 observed in cultured H-HSCs suggests that high levels of MEF2 proteins are associated with human liver fibrosis. We compared the levels of MEF2 proteins in freshly isolated H-HSCs from normal and cirrhotic human livers. In comparison to the low levels of MEF2A, C, and D proteins in H-HSCs from normal patients, the levels of MEF2 proteins were much higher (Figure 2) in H-HSCs isolated from cirrhotic livers, indicating that increased levels of MEF2s correlate well with the development of liver cirrhosis in humans.

### Regulation of MEF2 Expression by TGF-β1 in H-HSCs

**Figure 3:**
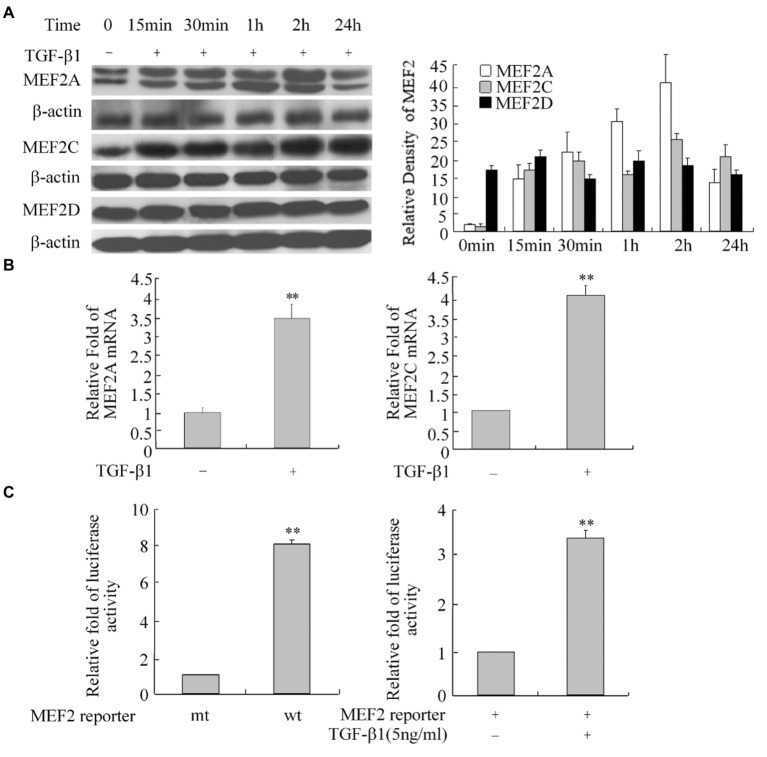
Increase in MEF2 expression during TGF-β1-induced activation of H-HSCs. **(A)** Regulation of MEF2 protein expression by TGF-β1. The levels of MEF2 proteins and β-actin at different time points following TGF-β1 (10 ng/ml) treatment were determined by western blot on day 7 in primary H-HSCs. The right graph represents quantification (*n* = 5). **(B)** TGF-β1-induced increase of MEF2 mRNA expression. Primary H-HSCs were treated with TGF-β1 for 2 h at day 7 in culture. MEF2 mRNAs was determined by RT-qPCR (*n* = 3, **p < 0.01 vs. without TGF-β1-induction). **(C)** Increase in MEF2-dependent gene transactivation activity after TGF-β1 treatment. Left panel, primary H-HSCs on day 5 in culture were transfected with a vector for the MEF2 luciferase reporter for 48 h. Right panel, primary H-HSCs on day 5 in culture were transfected with a vector for the MEF2 luciferase reporter for 46 h and treated with TGF-β1 (5 ng/ml) during the last 2 h. (wt, wild-type MEF2 luciferase reporter; mt, reporter with mutated MEF2 binding sites; *n* = 3). Luciferase activity was determined (*n* = 3, **p < 0.01, TGF-β1 vs. untreated control).

As a potent profibrogenic cytokine of HSCs, TGF-β1 is a key regulator of liver fibrosis (Friedman, 2008). To determine whether TGF-β1 induces MEF2 expression in H-HSCs, we cultured the H-HSCs as described in Figure 1 and determined the expression of MEF2 proteins following the addition of TGF-β1. Exposure of H-HSCs to TGF-β1 after 7 days in culture led to a robust increase in the levels of both MEF2A and MEF2C (Figure 3A). In contrast, MEF2D levels remained unchanged following TGF-β1 treatment. To correlate the increases in MEF2A and MEF2C protein expression, we determined their mRNA levels in primary H-HSCs cultured for 7 days, by RT-qPCR. Our data showed that TGF-β1 increased the mRNA levels of MEF2A and C (Figure 3B). We then examined the potential of MEF2 to activate gene transcription in H-HSCs using a sensitive MEF2-dependent reporter gene assay. The H-HSCs cultured for 7 days were transiently transfected with a luciferase reporter plasmid containing two copies of the MEF2 binding sites in the 5′-regulatory region of the luciferase gene. The same luciferase reporter with mutated MEF2 sites was used as the control. The cells were cotransfected with a plasmid for β–Gal as an internal control for transfection efficiency. Cultured H-HSCs showed high levels of MEF2-dependent luciferase activity (a nearly eight-fold increase in wild-type MEF2 reporter activity vs. mutant MEF2 reporter control) (Figure 3C, left graph). Interestingly, TGF-β1 further enhanced MEF2 activity (Figure 3C).

### Regulation of MEF2 and Human HSC Activation by TGFβ1-p38 MAPK

**Figure 4:**
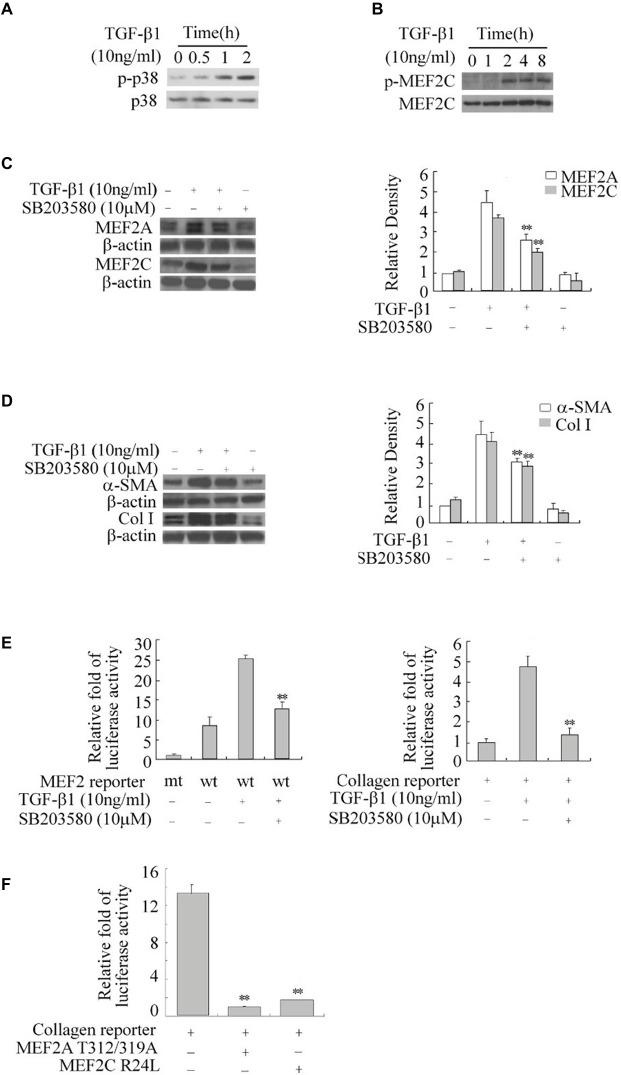
Regulation of the expression of MEF2, α-SMA, and collagen I by TGF-β1-p38 MAPK in HSCs. **(A)** Activation of p38 by TGF-β1. H-HSCs were treated with TGF-β1 for different lengths of time. Levels of phosphorylated p38 (p-p38) and total p38 were determined by western blot. **(B)** TGF-β1-induced phosphorylation of MEF2C. Levels of p-MEF2C and total MEF2C were determined by western blot. **(C)** Inhibition of TGFβ1-induced regulation of MEF2 by the p38 inhibitor. Primary H-HSCs were pretreated with SB203580 (p38 inhibitor) or vehicle for 24 h and treated with TGFβ1 during the last 2 h. MEF2A and MEF2C were determined by western blot. **(D)** Inhibition of TGFβ1 induced-α-SMA and collagen I expression by p38 inhibitor. The assays were performed as described in **(C)** (*n* = 3, **p < 0.01 TGF-β1-treated samples with SB203580 vs. without SB203580). **(E)** Inhibition of TGFβ1 induced MEF2 transactivation activity and collagen I reporter in H-HSCs. H-HSCs on day 5 in culture were transfected with mutated (mt) or wild type (wt) MEF2 reporter overnight, and then treated with TGFβ1 for 2 h with or without SB203580 pretreatment over 24 h. The luciferase assay was performed (*n* = 3; **p < 0.01 wild-type reporter induced by TGF-β1 with SB203580 treatment vs. without treatment). **(F)** Inhibition of collagen I reporter by blocking MEF2. The collagen I reporter gene assay was determined at day 7 in primary culture, 48 h after transfection with the co-expression of dominant negative MEF2 as indicated (*n* = 3, **p < 0.01 vs. controls.).

The p38 MAPK is downstream of the TGF-β1 signal and has been shown to directly phosphorylate and stimulate MEF2s in neurons (Mao et al., 1999; Liu et al., 2003). We tested the role of p38 MAPK in TGF-β1-induced regulation of MEF2s in HSCs. Western blot results showed that TGF-β1 causes a progressive increase in the levels of phosphorylated p38 (p-p38), indicating activation of p-38 (Figure 4A). This increase in p38 activity correlated well with enhanced phosphorylation of MEF2C at its p38 recognition site (Figure 4B). To confirm whether p38 regulates MEF2s in response to TGF-β1 in HSCs, we inhibited p38 MAPK with a specific inhibitor SB203580. Inhibition of p38 MAPK significantly attenuated the TGF-β1-induced increase in MEF2 levels and activity (Figure 4C). Consistently, inhibition of p38 MAPK also downregulated the TGF-β1-induced activation of the collagen I promoter and greatly attenuated the TGF-β1-induced increase in the levels of collagen I and α-SMA (Figures 4D,E).

To show that p38-mediated regulation of MEF2s is involved in collagen and α-SMA expression, we tested the effects of dominant negative MEF2A, which carries mutations at its p38 phosphorylation sites and does not respond to p38 MAPK signaling in H-HSCs. Overexpression of this MEF2A mutant significantly inhibited the activity of the collagen I promoter (Figure 4F). Similarly, overexpression of MEF2C R24L, which inhibits endogenous MEF2s *via* a different mechanism, also reduced collagen promoter activity.

### Effects of Antifibrogenic Agent, SA-B, on MEF2

**FIGURE 5:**
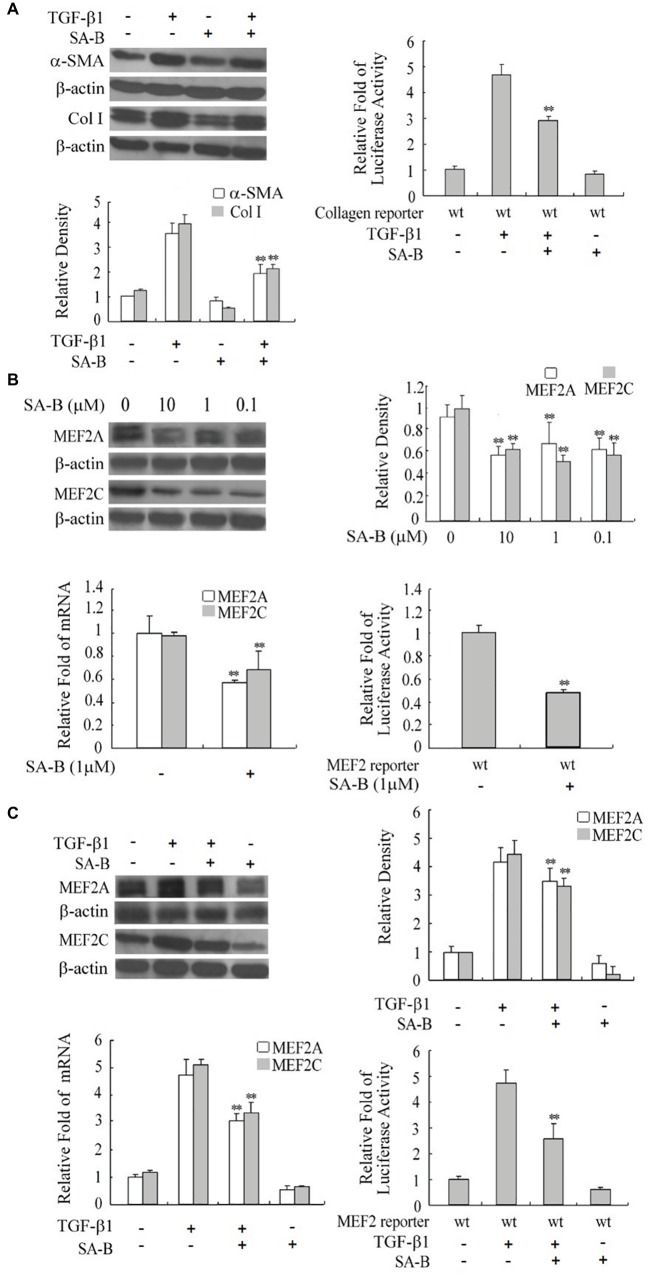
Reduction of the expression of MEF2s and markers of H-HSC activation by SA-B. **(A)** Expression of Col I and α-SMA in H-HSC treated with SA-B or TGF-β1. Day 5 H-HSCs were treated with SA-B (1 μM) for 48 h, and then treated with TGF-β1 (10 ng/ml) for 2 h (*n* = 3; **p < 0.01; Col I and α-SMA induced by TGF-β1 pretreated with SA-B vs. non-treated). The collagen luciferase reporter gene assay was determined. Relative fold change in luciferase activity is shown (*n* = 3, **p < 0.01, wild-type reporter induced by TGF-β1 with SA-B treatment vs. without SA-B treatment). **(B)** Expression of MEF2 in H-HSC treated with SA-B. Day 5 H-HSCs were treated with SA-B at different concentrations for 48 h. Protein level was analyzed by western blot (top left panel). Densitometry values of MEF2 relative to β-actin are shown (top right panel) (*n* = 3; **p < 0.01 vs. non-treated with SA-B). The MEF2 mRNA expression was determined by RT-qPCR following SA-B (1 μM) treatment as described above (bottom left panel) (*n* = 3, **p < 0.01, with vs. without SA-B). The MEF2-dependent luciferase reporter gene assay was determined (bottom right panel: *n* = 3; **p < 0.01, wild-type reporter treated with SA-B vs. without SA-B). **(C)** Expression and activity of MEF2 treated with SA-B or TGF-β1. Day 5 H-HSCs were treated with SA-B (1 μM) for 48 h and then treated with TGF-β1 (10 ng/ml) for 2 h. Protein level of MEF2 was determined by western blot (top left panel) (*n* = 3; **p < 0.01, TGF-β1-treated samples with SA-B vs. without SA-B). MEF2 mRNA expression and reporter activity assay was determined (bottom panel) (*n* = 3; **p < 0.01, TGF-β1 treated samples pretreated with SA-B vs. without SA-B).

The proliferation and activation of rat HSCs are effectively inhibited by SA-B (Lv et al., 2010). We confirmed that treating H-HSCs with SA-B reduces the TGF-β1-induced increase of α-SMA and collagen I production. Similarly, SA-B also reduced collagen reporter activity in H-HSCs at day 5 in culture (Figure 5A).

To verify if SA-B regulates HSC activation *via* downregulation of MEF2, we treated H-HSCs with SA-B and determined its effect on MEF2 proteins. The SA-B evidently caused a decline in the levels of MEF2A and C protein and mRNA. Consistently, SA-B also reduced MEF2-dependent luciferase reporter activity (Figure 5B). We tested whether the effects of TGF-β1 on MEF2 were inhibited by SA-B in H-HSCs. The SA-B inhibited the TGF-β1-induced increase in MEF2 mRNA and protein levels and attenuated TGF-β1-mediated activation of MEF2 activity (Figure 5C). These results suggest that SA-B is capable of inhibiting TGF-β-induced activation of MEF2 at multiple levels in H-HSCs.

### Effects of SA-B on MEF2A and α-SMA in the Rat Liver, Following DMN-Induced Fibrosis

**Figure 6:**
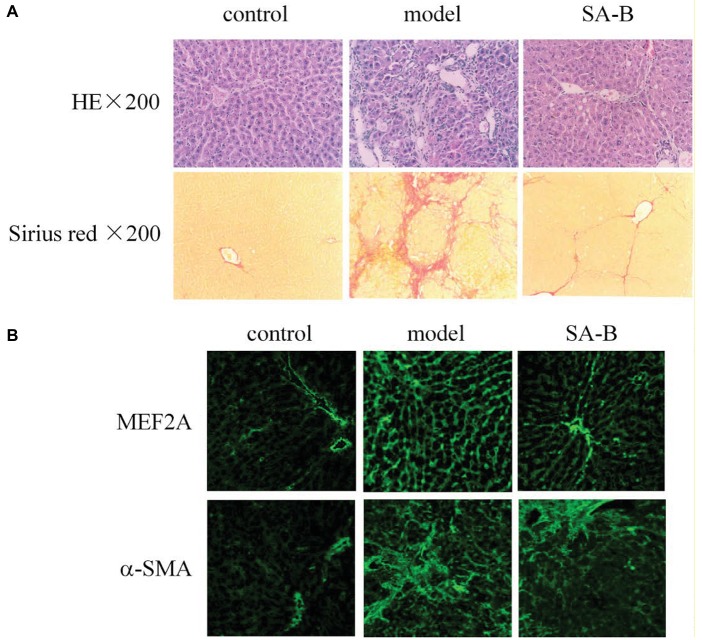
Inhibition of MEF2 and α-SMA expression by SA-B in the rat liver following DMN-induced fibrosis. **(A)** DMN-induced rat liver fibrosis. Sections were stained with H&E and Sirius red. **(B)** MEF2 and α-SMA expression in normal control, DMN-induced fibrosis, and SA-B-treated livers. Proteins were determined by immunofluorescence.

Our results above showed that HSCs from patients with cirrhosis express higher levels of MEF2 proteins. In addition, SA-B is capable of inhibiting the expression of MEF2 stimulated by TGF-β1 in H-HSCs. To correlate MEF2 levels and fibrogenic response *in vivo*, we examined the expression of MEF2A and α-SMA in the rat liver using a well-established model of DMN-induced fibrosis. Injection of DMN induced liver fibrosis and cirrhosis in the rat (Figure 6A). Compared with the normal rat liver control, the expressions of MEF2A and α-SMA were significantly induced after treatment with DMN for 4 weeks (Figure 6B). The levels of MEF2A and α-SMA were evidently reduced by SA-B, which correlates well with evidence of reduced Sirius red staining.

## Discussion

Most previous studies on the complex mechanisms involved in the development of liver fibrosis in humans have been conducted primarily using HSCs isolated from animals (Jiao et al., 2016; Vilaseca et al., 2017; Du et al., 2018). However, whether the molecular mechanisms identified in animal cells can be directly translated to human HSCs, particularly, primary human HSCs, is unclear. We addressed this question by testing the role and regulation of the MEF2s, which are transcription factors that have been identified to play a role in rat HSCs (Wang et al., 2004). Our current studies showed that MEF2s function as downstream effectors of TGF-β1 signals to regulate human HSC activation and the fibrogenic response. We identified p38 MAPK as the major mediator activated by TGF-β1 to regulate MEF2 in H-HSCs during liver fibrosis. The MEF2 proteins show higher levels in the HSCs isolated from cirrhotic patients than those from control patients. Moreover, SA-B, a herbal extract with known clinical antifibrogenic effects in liver fibrosis (Liu et al., 2002a), attenuated TGF-β1-induced activation of MEF2s and MEF2-dependent collagen I gene expression in H-HSCs and in a liver fibrosis model in the rat. Given that the process of liver fibrosis is required for the development of cirrhosis, our results show that MEF2 participates in the development of human liver fibrosis and may contribute to the pathogenesis of cirrhosis.

Although transcriptional regulation of MEF2 in rat HSC activation has been demonstrated (Wang et al., 2004), the manner in which this class of factors is regulated by profibrogenic cytokines has not been established prior to our study. Our studies using human primary HSCs revealed differential regulation of various isoforms of MEF2s and extended findings in rat HSCs. We showed that although plating H-HSC MEF2A led to activation of H-HSCs and an increase in the protein levels of MEF2A, C, and D, TGF-β1 treatment of H-HSCs for 7 days enhanced only the expression of MEF2A and C but not MEF2D. The reason for this difference in MEF2 isoform response is presently unknown. As MEF2 proteins form either homodimers or heterodimers to regulate gene expression (Tang et al., 2005), it is not clear whether MEF2D (not induced by TGF-β1) may contribute to the overall activity of MEF2s in cells.

Our data defined TGF-β1 as a stimulus of MEF2 expression during the activation of H-HSCs. Several pathways have been shown to mediate the TGF-β1 signal in various cell types (Friedman, 2008). Previous studies have shown that p38 MAPK directly phosphorylates and activates MEF2s in response to survival signals in neurons (Mao et al., 1999). Our current findings suggest that p38 MAPK plays a major role in mediating TGF-β1-induced activation of MEF2s. It is interesting to note that although inhibition of p38 MAPK clearly attenuated TGF-β1-induced increase in the MEF2 protein, this inhibition was incomplete. Whereas p38 MAPK is known to directly interact with MEF2 proteins, TGF-β1 also affects MEF2 function by increasing the levels of MEF2 mRNAs. These data are consistent with the possibility that other downstream mediators of the TGF-β1 signal may also participate in regulating MEF2s. As MEF2s are known to interact with the Smad family of proteins in muscle cells (Quinn et al., 2001), it would be interesting to test whether mediators other than p38, such as Smad or repressors of the TGF-β1 signal, may participate in the regulation of MEF2s, particularly at the level of transcription (Gong et al., 2003).

Previous studies have described the antifibrogenic effects of SA-B in rat HSCs. SA-B suppressed HSC proliferation concentration dependently, inhibited soluble type I collagen secretion, and decreased the matrix collagen deposition. SA-B at 1 and 10 μmol/L decreased the cell active TGF-β1 secretion by 63.3% and 15.6% of the control (Liu et al., 2002b). The mechanisms by which SA-B exerts its anti-fibrosis effects are not entirely clear. Our data suggest that SA-B is a potent antifibrogenic agent against the culture-induced activation of primary human HSCs. In addition, SA-B directly antagonizes the TGF-β1-induced fibrogenic response in human HSCs. Moreover, SA-B not only reduces the increase in MEF2 mRNA levels but also attenuates the accumulation of MEF2 proteins. Our studies suggest that antagonizing TGF-β1-induced activation of MEF2 activity at both protein and RNA levels may account in part for the anti-fibrosis effects of SA-B.

## Conclusion

The MEF2 transcription factor is stimulated by TGF-β1 in H-HSCs. Antagonizing TGF-β1-induced activation of the MEF2 signaling pathway may account in part for the anti-fibrosis effects of SA-B.

## Author Contributions

WZ and JP performed the experiments and wrote the manuscript. YZ assisted with the animal experiments. GC assisted with histomorphological experiments. LX designed the experiments and interpreted data.

### Conflict of Interest Statement

The authors declare that the research was conducted in the absence of any commercial or financial relationships that could be construed as a potential conflict of interest.

## Abbreviations

DMN: dimethylnitrosamine
ERK: extracellular signal-regulated kinase 1
GFAP: glial fibrillary acidic protein
H-HSCs: human hepatic stellate cells
p38 MAPK: p38 mitogen-activated protein kinase
MEF2: myocyte enhancer factor 2
SA-B: salvianolic acid B
α-SMA: α-smooth muscle actin
TGF-β1: transforming growth factor β1.
